# Editorial: Clinical application of machine learning methods in psychiatric disorders

**DOI:** 10.3389/fpsyt.2023.1209615

**Published:** 2023-05-31

**Authors:** Xiaozheng Liu, Zhi Xu, Weikai Li, Zhen Zhou, Xize Jia

**Affiliations:** ^1^Department of Radiology, Second Affiliated Hospital and Yuying Children's Hospital of Wenzhou Medical University, Wenzhou, China; ^2^Department of Psychosomatics and Psychiatry, Zhongda Hospital, School of Medicine, Southeast University, Nanjing, China; ^3^School of Mathematics and Statistics, Chongqing Jiaotong University, Chongqing, China; ^4^Department of Radiology, Center for Biomedical Image Computing and Analytics, University of Pennsylvania, Philadelphia, PA, United States; ^5^School of Psychology, Zhejiang Normal University, Hangzhou, China

**Keywords:** machine learning, psychiatric disorders, clinical application, natural language processing, functional magnetic resonance imaging (fMRI)

## Introduction

With the increasing prevalence of psychiatric disorders, there has been a significant increase in the demand for effective psychiatric disorder treatments in recent years. However, the complexity of neuronal degeneration and the heterogeneity of patients make early diagnosis and treatment of these disorders difficult. To meet these challenges, scientists, clinicians, and patients can benefit from the application of machine learning theories and algorithms. Machine learning, which includes methods for feature extraction, selection, and classification, has demonstrated significant benefits in the pathological analysis of psychiatric disorders (1). These methods can learn features from brain neuroimaging data and adapt to data variation, thereby improving the reliability, performance, and accuracy of disease-specific diagnostic systems. Furthermore, machine learning can accurately assess the conditions of patients. With the use of cutting-edge machine learning algorithms, clinical diagnosis, and clinical interventions, along with clinical neuroimaging data of the brain, this Research Topic aims to incorporate theoretical and technological innovations and assess the performance of machine learning in clinical studies on psychiatric disorders.

## Machine learning methods in psychiatric disorders

Deep learning-based natural language processing techniques were applied to assess depressive symptoms in clinical interviews. The F1 score (a measure of model performance, harmonic mean of accuracy, and recall) was 0.719 when classifying the four-level severity of depression, and 0.890 when identifying the presence of depressive symptoms (Li et al.). Multidimensional speech feature diagnosis and evaluation system (MSFDA) combining multidimensional speech features and deep learning in the auxiliary diagnosis of major depressive disorder in children and adolescents. The sensitivity (92.73% vs. 76.36%) and specificity (90.91% vs. 85.45%) of the MSFDA system were significantly higher than those of HAMD-24. The area under the curve of the MSFDA system is also higher than that of HAMD-24. The difference between the two groups was statistically significant (*p* < 0.05), and the diagnostic accuracy was higher in both groups. In addition, the diagnostic efficiency of the MSFDA system is higher than that of HAMD-24 in terms of Youden index, diagnostic accuracy, likelihood ratio, diagnostic odds ratio and predictive value (Luo et al.).

The combination of artificial intelligence and imaging data to guide clinical diagnosis and intervention is a major direction for future clinical research. Venkatapathy et al. present an integrated model for resting-state functional MRI data analysis and graph convolution networks based on graph theory. For the classification of patients with major depressive symptoms and healthy controls, the ensemble models achieved 71.18% upsampling accuracy and 70.24% downsampling accuracy. When comparing patients with first-episode major depressive symptoms to those with recurrent major depressive symptoms, the accuracy of upsampling was 77.78% and the accuracy of downsampling was 71.96% (Venkatapathy et al.). A multimodal MRI image-based imagingomics study predicted prognostic outcome of stroke with an accuracy of 0.831, sensitivity of 0.739, specificity of 0.902, F1 score of 0.788 and area under the curve of 0.902 (Yu et al.). Resting-state-based amplitude of low-frequency fluctuation and support vector machine models help distinguish schizophrenia patients from healthy controls (Gao et al.).

Peripheral blood is easy to obtain and less invasive, making it an ideal specimen for clinical trials. Lymphocyte subpopulation-based features help distinguish bipolar depression from major depressive disorder with an accuracy of >90% (Su et al.). The accuracy of the peripheral non-enzymatic antioxidant combined with the xGboost model for differentiating bipolar disorder from major depressive disorder was 0.849 and for differentiating bipolar disorder with depressive episodes from major depressive disorder was 0.899 (Gong et al.).

## Conclusion

Each paper focuses on a different but equally important aspect of clinical application of machine learning methods in psychiatric disorders. We believe that by presenting and highlighting the latest novel and emergent machine learning technologies, implementations, and applications relating to psychiatric disorders, we will raise awareness in the scientific community. Finally, we'd like to thank all of the authors who contributed to this Research Topic with their research. We would also like to thank the many experts in the field who participated in the review process and offered helpful suggestions to the authors to improve the articles' contents and presentations.

## Author contributions

XL were major contributors in writing the manuscript. All authors read and approved the final manuscript.
